# Detecting Image Splicing Using Merged Features in Chroma Space

**DOI:** 10.1155/2014/262356

**Published:** 2014-01-16

**Authors:** Bo Xu, Guangjie Liu, Yuewei Dai

**Affiliations:** School of Automation, Nanjing University of Science & Technology, Nanjing 210094, China

## Abstract

Image splicing is an image editing method to copy a part of an image and paste it onto another image, and it is commonly followed by postprocessing such as local/global blurring, compression, and resizing. To detect this kind of forgery, the image rich models, a feature set successfully used in the steganalysis is evaluated on the splicing image dataset at first, and the dominant submodel is selected as the first kind of feature. The selected feature and the DCT Markov features are used together to detect splicing forgery in the chroma channel, which is convinced effective in splicing detection. The experimental results indicate that the proposed method can detect splicing forgeries with lower error rate compared to the previous literature.

## 1. Introduction

Image splicing is a forgery manner to copy and paste regions within separate image sources, usually carried out by digital image editing tools such as Photoshop. It is often used as an initial step of photomontage, which is very popular in digital image content editing. The splicing tampered image could be used in news reports, photography contest, key proof in the academic papers, and so on, which could bring certain negative influences. As a result, it is an important issue to develop reliable splicing detection methods.

In the forgery process, the manually introduced transitions of edges and corners are different from those in the natural images. The differences are commonly described by the inconsistency and abnormality, and they are used for splicing detection. Farid [1] discussed how to detect unnatural higher-order correlations introduced into the signal by the tampering process based on bispectral analysis. In the frequency domain, a “natural” signal usually has weak higher-order statistical correlations, and certain “unnatural” correlations will be introduced if this signal is passed through a nonlinearity (which would almost surely occur in tampering). Based on it, Ng et al. [2] proposed a method to detect the abrupt splicing discontinuity using the bicoherence features. They studied the effects of image splicing on magnitude and phase characteristics of bicoherence (the normalized bispectrum). The difference between means of magnitudes of a test image's bi-coherence and its estimating authentic vision's bi-coherence and the difference between negative phase entropy of those two were used as features. Gopi et al. [3, 4] proposed to detect forgeries using an artificial neural network, independent component analysis (ICA), and autoregressive coefficients. In [5, 6], Hsu and Chang proposed a method based on camera response function estimated from geometry invariants. Wang and Zhang [7] employed the Sobel edge detector, derivative operation, and Hough transform to detect image splicing. Lint et al. [8] proposed a method based on computing the inverse camera response functions by analyzing the edges in different patches of the image and verifying their consistency.

Shi et al. proposed to use statistics of 2D phase congruency [9], a natural image model [10], and Hilbert-Huang transform [11] to separate spliced images from authentic ones. They believed that on one hand, steganography and splicing had different goals and strategies causing different statistical artifacts on images, on the other hand, both of them made the touched (stego and spliced) image different from corresponding original ones. Therefore, they built a natural image model using steganalysis features including statistical moments of characteristic functions and Markov transition probabilities from image 2D array and multisize block discrete cosine transform 2-D array. Wang et al. [12] proposed an image splicing detection method based on GLCM of the edge image in the chroma space. Zhang et al. [13] proposed a splicing detection scheme based on moment features extracted from the discrete cosine transform and image quality features. Dong et al. [14] proposed a method by analyzing the discontinuity of image pixel correlation and coherency caused by splicing, and they also proposed to detect image splicing in chroma space [15], which is claimed to be very efficient. Bayram et al. [16] fused several single tools together to detect tampering. The feature vectors BSM, IQM, and HOW, which were initially proposed for steganalysis, were used in this paper, and SFFS (sequential forward floating search) is used for feature selection.

Existing methods exploit features from various respects to detect splicing forgeries. Recently, Fridrich and Kodovský [17] proposed to build plenty of submodels as rich model to detect image steganography, which achieved good results. The SRM exploits the spatial correlations of neighborhood pixels from the image residuals which are hard to be preserved during image steganography. It is believed that the case would be the same for image splicing. In this paper, the image splicing detection method based on SRM is studied. AT first, the performance of the submodels is evaluated and analyzed by the detailed experimental results in the luminance channel. Moreover, one selected submodel with feature from DCT is used to detect splicing forgery in chroma channel.

The rest of the paper is organized as listed in following. The SRM is described in Section 2.  Section 3 discusses the detection method. And experiments are given in Section 4. In Section 5, a conclusion is drawn to the whole paper.

## 2. Feature Discriptions

In [17], Fridrich and Kodovský proposed a general methodology for steganalysis of digital images based on the concept of the SRM consisting of a large number of diverse submodels. The submodels consider various types of relationships among neighboring samples of noise residuals obtained by linear and nonlinear filters with compact supports.

### 2.1. SRM

The SRM captures a large number of different types of dependencies among neighboring pixels. By employing the submodels, better results can be achieved beyond enlarging a single model, which is unlikely to produce good results as the enlarged model will have too many underpopulated bins. By forming a model by merging many smaller submodels, this problem can be avoided.

The main formation processes of SRM are residuals, truncation, and quantization and cooccurrence matrix. The description of all residuals is given here, for other detail description, please refer to [17]. For example, in the first order residual *R*_*ij*_ = *X*_*i*,*j*+1_ − *X*_*i*,*j*_, the central pixel *X*_*i*,*j*_ is predicted as its immediate neighbor, ${\hat{X}}_{ij}=X_{i,j+1}$, while the predictor in the second order residual *R*_*ij*_ = *X*_*i*,*j*−1_ + *X*_*i*,*j*+1_ − 2*X*_*i*,*j*_ assumes that the image is locally linear in the horizontal direction, $2{\hat{X}}_{i,j}=(X_{i,j-1}+X_{i,j+1})$.

#### 2.1.1. Residual Classes

The residuals are divided into six classes depending on the central pixel predictor they are built from. The classes will be mentioned by the following descriptive names: 1st, 2nd, 3rd, SQUARE, EDGE3 × 3, and EDGE5 × 5. The predictors in class “1st” estimate the pixel as the value of its neighbor, while those from class “2nd” (“3rd”) incorporate a locally linear (quadratic) model. The class “SQUARE” makes use of more pixels for the prediction. The class “EDGE3 × 3” predictors, derived from the 3 × 3 square kernel S3a, were included to provide better estimates at spatial discontinuities (edges).

The larger 5 × 5 predictor in S5a was obtained as a result of optimizing the coefficients of a circularly-symmetrical 5 × 5 kernel. The “EDGE5 × 5” residuals E5a–E5d are built from S5a in an analogical manner as E3a–E3d are built from S3a.

#### 2.1.2. Residual Symmetries

Each residual exhibits symmetries that will later allow us to reduce the number of submodels and make them better populated. If the residual does not change after being computed from the image rotated by 90 degrees, it is nondirectional; otherwise, it is directional. For instance, 1a, 1b, 2a, 2e, and E3c are directional while 1e, 2b, 2c, S3a, and E3d are nondirectional. Two cooccurrence matrices are computed for each residual, one for the horizontal scan and the other for the vertical scan. We call a residual *hv*-symmetrical if its horizontal and vertical cooccurrences can be added to form a single matrix (submodel) based on the argument that the statistics of natural images do not change after rotating the image by 90 degrees.

#### 2.1.3. Syntax

The syntax of the names follows this convention:

(1) $$\begin{array}{c} \text{name}=\left\{\text{type}\right\}\left\{f\right\}\left\{\sigma \right\}\left\{\text{scan}\right\}, \end{array}$$

where type ∈ {spam, minmax}, *f* is the number of filters, *σ* is the symmetry index, and the last symbol scan ∈ {*∅*, *h*, *v*} may be missing (for *hv*-symmetrical residuals) or it is either *h* or *v*, depending on the cooccurrence scan that should be used with the residual. For more detailed descriptions and definitions of the SRM, please refer to [17].

### 2.2. Markov Matrices in DCT

**Figure 1:**
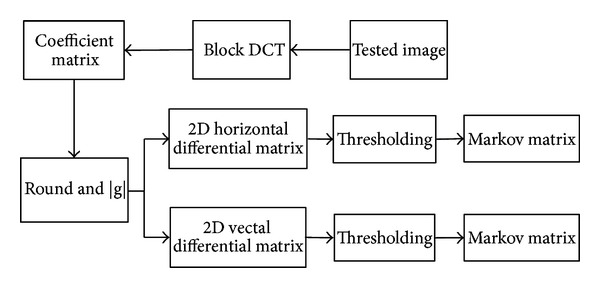
Feature extraction in DCT of Chrominance.

Splicing operation changes the local relationships in pixel neighborhood, and Markov process could be used to model the changes, which is commonly used in image processing. In this paper, the Markov feature is extracted in DCT of Chrominance space. As shown in Figure 1, the test image is transformed using 8 × 8 block DCT at first, and after preprocessing to the coefficient matrix, the 2D differential matrices and probability transition matrices are obtained using the following (Equations (2)–(3)):

(2) CMv(i,j)=(∑m=1M−2∑n=1N−2[δ(F(m,n)=i,F(m,n+1)=j)]) ×(∑m=1M−2∑n=1N−2δ(F(m,n)=i))−1,CMv(i,j)=(∑m=1M−2∑n=1N−2[δ(F(m,n)=i,F(m+1,n)=j)]) ×(∑m=1M−2∑n=1N−2δ(F(m,n)=i))−1,CMd(i,j)=(∑m=1M−2∑n=1N−2[δ(F(m,n)=i, F(m+1,n+1)=j)]) ×(∑m=1M−2∑n=1N−2δ(F(m,n)=i))−1,

where *i*, *j* ∈ [0,8], and

(3) $$\begin{array}{c} \delta \left(x=i,y=j\right)=\{\begin{array}{cc} 1, & \text{if}\;x=i\;\;\text{and}\;y=j,\\ 0, & \text{otherwise}. \end{array} \end{array}$$

If the added splicing image block is regarded as the additive noise to the original image, the calculated probability transition matrices would reveal the clue of splicing.

The dimension of the feature vector is adjustable by setting threshold T. In this paper, T is set to 3, and the dimension of DCT feature is 98.

## 3. Detection Method

The SVM is employed in the detection procedure. The LibSVM is introduced and profiled at first, followed by the training and testing steps.

### 3.1. LIBSVM

LIBSVM is a popular machine learning tool [16]. In this paper, the RBF kernel of LibSVM is employed, and *C* and gamma are automatically selected using fivefold cross-validation.

As different feature sets will be merged together in the detection algorithm, feature normalization is a necessary step before training and testing. The specific scaling method is very simple. Firstly, the mean and standard deviation for each feature set are calculated. Secondly, by subtracting the mean in each feature and dividing the values of each feature by its standard deviation, the normalized features are obtained. In favor of making comparison, all the training and testing processing are done after the feature normalization.

### 3.2. Training Steps

The detection method consists of the following steps.

1. Read an image from training image set and label it, 0 for authenticate image and 1 for spliced image. Covert it into 8 bit gray scale image if necessary.
2. Let *q* = 1, and compute the feature of submodel *s*{*i*} as described in Section 2.
3. Repeat 1-2 until all the images from the training image set have been processed.
4. Train the models for individual submodel or merged ones using LIBSVM as described in the previous parts.

### 3.3. Detection Steps

1. Read an image from the testing image set. Covert it into 8 bit gray scale image if necessary.
2. Let *q* = 1, and compute its feature by the individual submodel or the merged ones as the training steps.
3. Test the feature using the corresponding trained SVM.
4. Repeat steps (1)–(3) until all images have been tested.

## 4. Experiments

According to the detection method discussed in Section 3, the experiments are performed. At first, the general detection performance of the individual submodel is tested on both image datasets in luminance channel. And then, one submodel is chosen for the chroma channel experiment and comparison to the state of the art result is given.

### 4.1. Experiment Setup

#### 4.1.1. Image Dataset

In the experiment, two splicing image datasets are used. The Columbia uncompressed image splicing detection evaluation dataset [5] is commonly used as a benchmark. The database contains totally 183 authenticate images and 180 spliced images. The image sizes range from 757 × 568 to 1152 × 768 and are all uncompressed. The image detection evaluation database (CASIA TIDE) v2 consists of 7,491 authentic and 5,123 sophisticatedly tampered color images of different sizes varying from 240 × 160 to 900 × 600. This database is with larger size and more realistic and challenging tampered images with complex splicing as well as blurring. The authentic images are collected from the Corel image dataset, websites, and so on. The tampered images are generated with resizing, rotation, or other distortion, and postprocessing (such as blurring) is performed after cut-and-past operation, which make the dataset more realistic and challenging.

#### 4.1.2. Experiment Settings

**Figure 2:**
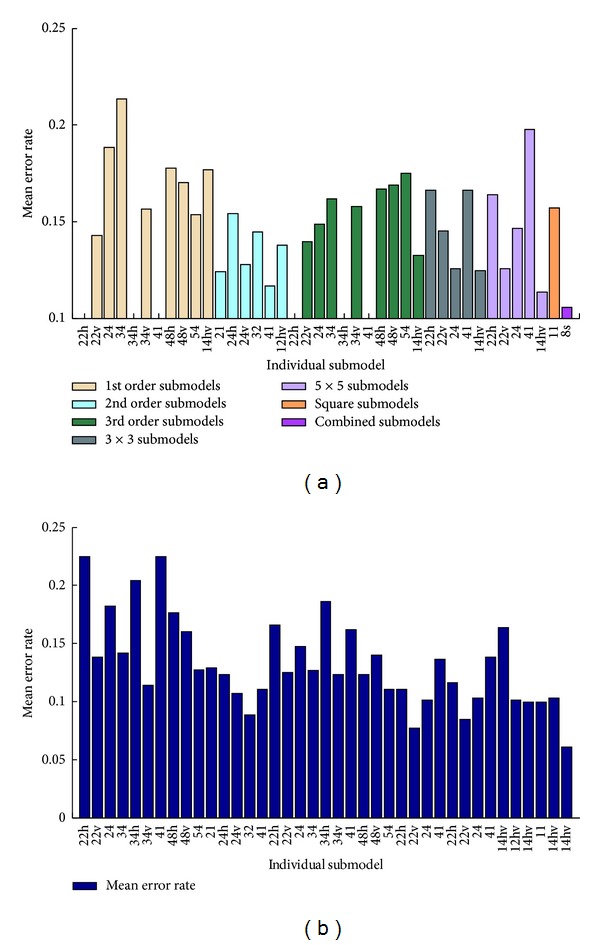
The mean errors of individual submodels for (a) Columbia dataset, (b) CASIA V2 dataset. Blank bars represent that the mean errors of the models are not available.

The detection results of the experiment are evaluated by the detection error with the form as

(4) $$\begin{array}{c} P_{E}=\frac{1}{2}\left(P_{\text{FA}}+P_{\text{MD}}\right), \end{array}$$

where *P*_FA_ and *P*_MD_ are the probabilities of false alarm and missed detection within the model whose parameters *C* and *γ* are both obtained from the cross validation. We repeat the experiments of each model for 10 times and take the average of them as the final results, which is shown in Figure 2.

### 4.2. Analysis of Individual Models in Luminance Channel

Figure 2 shows the mean errors of the valid individual models, in both datasets. Generally speaking, all the submodels are useful in the splicing detection, and the top results are observed by using higher order features, the second order submodel, the EDGE3 × 3 models, and the EDGE5 × 5 models, rather than first order submodel.

**Figure 3:**
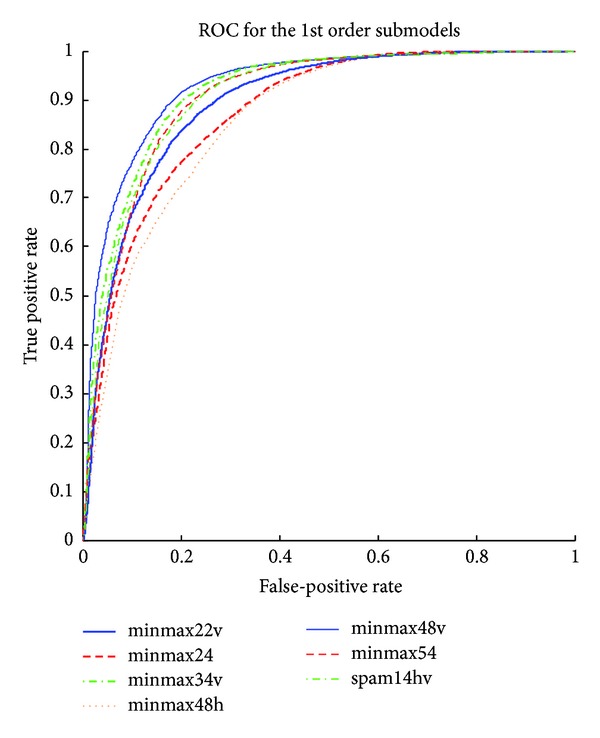
ROC curves for first order features.

**Figure 4:**
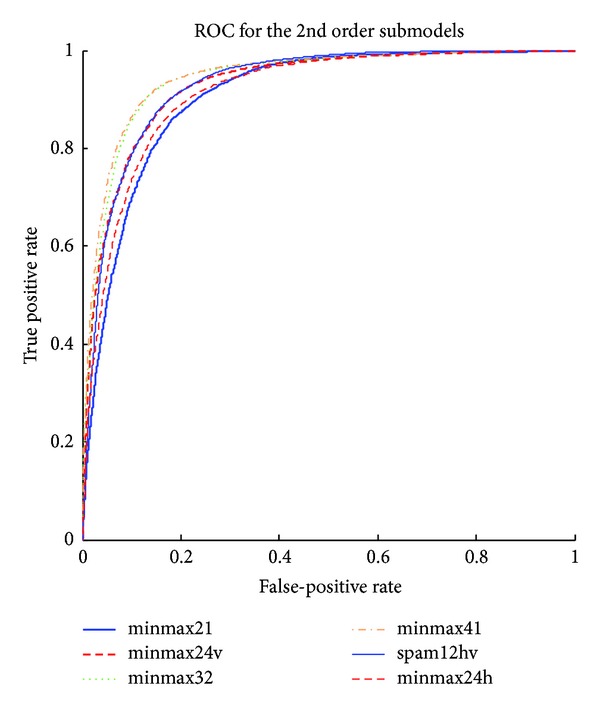
ROC curves for second order features.

**Figure 5:**
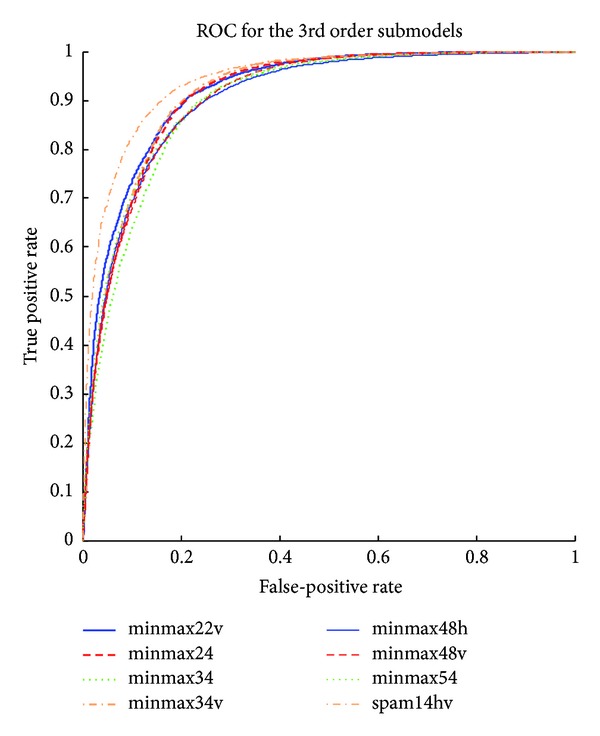
ROC curves for third order features.

**Figure 6:**
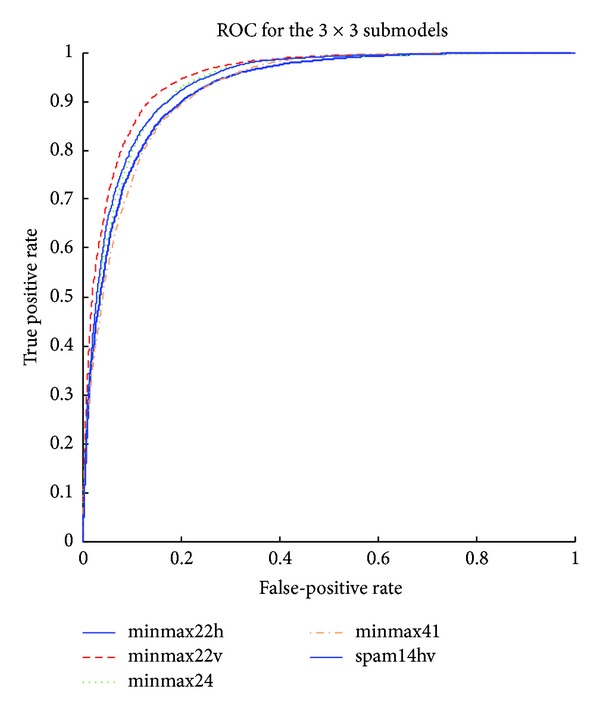
ROC curves for EDGE3 × 3 features.

**Figure 7:**
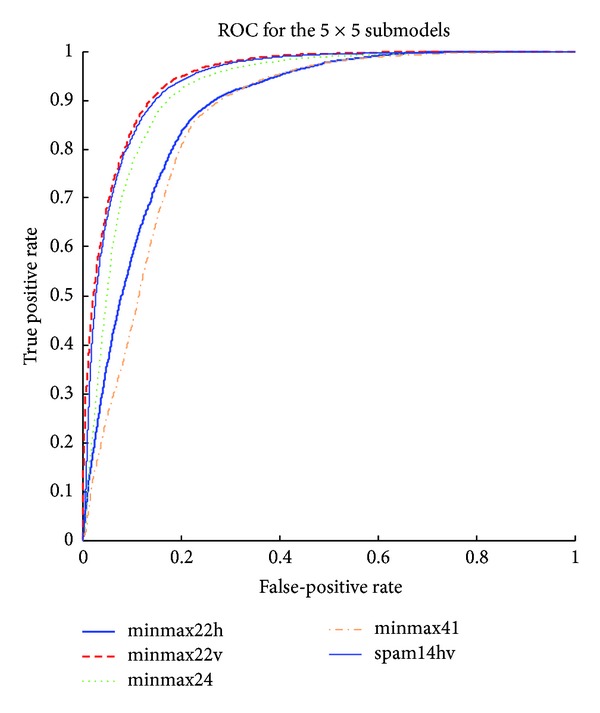
ROC curves for EDGE5 × 5 features.

**Figure 8:**
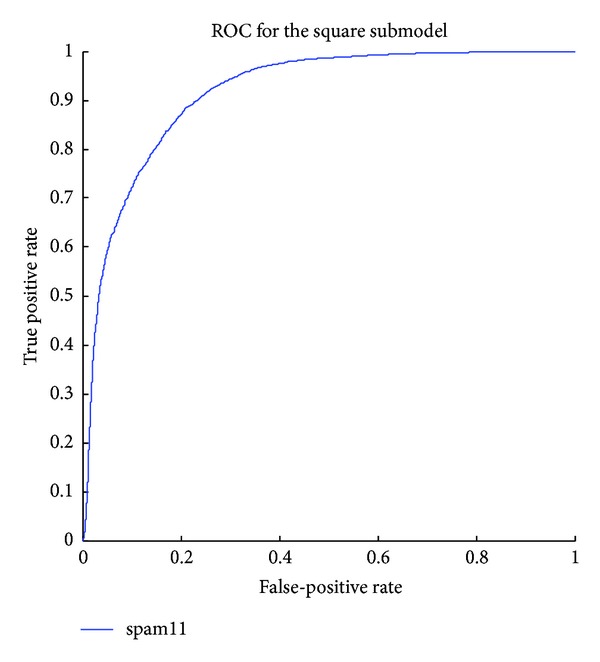
ROC curves for the square features.

**Figure 9:**
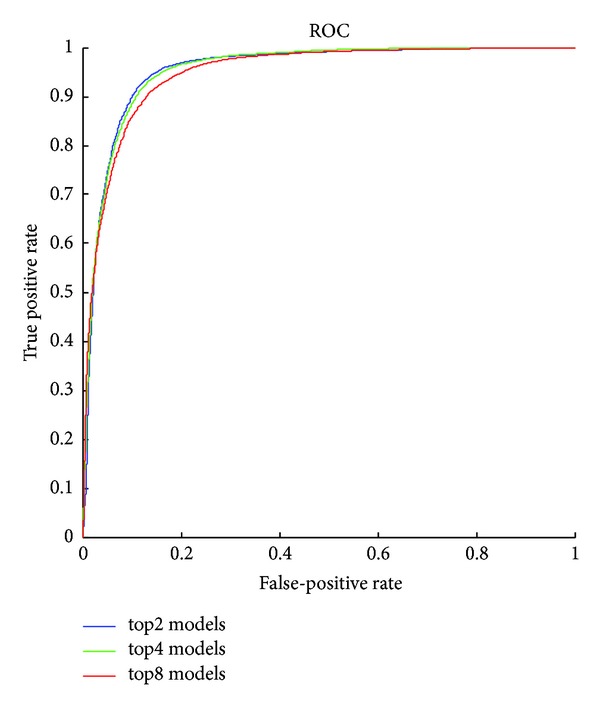
ROC curves for combined features.

The detailed results of the submodels are listed in Tables 1, 2, 3, 4, 5 and 6 and the ROC curves of them are given in Figures 3, 4, 5, 6, 7, and 8. As for the merged models, the top eight submodels with best detection results are highlighted in boldface in Tables 2–5, the top four submodels are highlighted in boldface and italic, and the top 2 submodels are s5 × 5_spam14hv and s2_minmax41. The ROC curve of them is given in Figure 9.

### 4.3. Experiment in Chroma Channel

**Figure 10:**
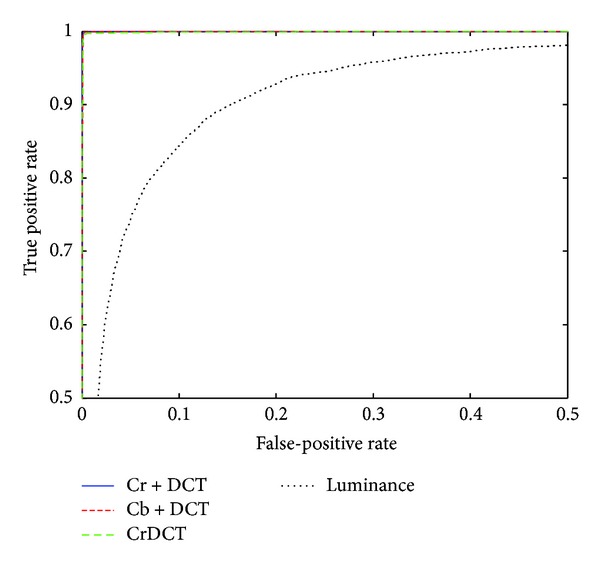
ROC curves for detection results of YCbCr channel on the CASIA v2 dataset.

Based on the results from the previous section, a submodel from EDGE3 × 3, s3 × 3_14hv, is selected for further experiment. It is shown in [15] that chorma channel is more effective than luminance channel in detecting image splicing forgeries. The CASIA V2 dataset, which is larger and more challenging, is used for the experiment. The results are listed in Table 7. We can find that the *P*_FA_ drops about 15% when changing feature from Y channel to Cb, Cr channels, and the *P*_*E*_ drops about 13%. The SRM feature from Cb channel combined with the DCT feature from Cr channel achieved the best *P*_*E*_. The ROC curve is shown in Figure 10.

## 5. Conclusions

SRM exploits plenty of statistical features from neighbors of image spatial domain, which could capture abnormality caused by steganography. In this paper, we try to detect the splicing introduced abnormality using SRM. The 39 submodels are exploited to detect image splicing in luminance channel at first. Then, the submodel s 3 × 3_14hv is selected and merged with DCT feature to detect splicing in chorma channel on CASIA V2 dataset, which is larger and more challenging. The experimental results indicate that the proposed method can detect splicing forgeries with much higher accuracy than in luminance channel.

In the near future, we will try to study the nonlearning based detention method in chorma channel, which could be helpful in revealing the underlying mechanic of the method, and could be better for practical application.

## Figures and Tables

**Table 1:** Detection results of the first order submodels.

Model	*P*_FA_	*P*_MD_	*P*_*E*_ (total error)
s1_minmax22h	1	0	0.5
s1_minmax22v	0.1401	0.1457	0.1429
s1_minmax24	0.1549	0.2219	0.1884
s1_minmax34	0.2097	0.2175	0.2136
s1_minmax34h	1	0	0.5
s1_minmax34v	0.1334	0.1799	0.1567
s1_minmax41	1	0	0.5
s1_minmax48h	0.1556	0.1996	0.1776
s1_minmax48v	0.1488	0.1915	0.1702
s1_minmax54	0.1286	0.1786	0.1536
s1_spam14hv	0.1661	0.1876	0.1768

**Table 2:** Detection results of the second order submodels.

Model	*P*_FA_	*P*_MD_	*P*_*E*_ (total error)
**s2_minmax21**	**0.1233**	**0.1247**	**0.124**
s2_minmax24h	0.1276	0.181	0.1543
**s2_minmax24v**	**0.1178**	**0.138**	**0.1279**
s2_minmax32	0.13	0.1591	0.1446
***s2_minmax41***	***0.1122***	***0.1214***	***0.1168***
s2_spam12hv	0.1193	0.1565	0.1379

**Table 3:** Detection results of the third order submodels.

Models	*P*_FA_	*P*_MD_	*P*_*E*_ (total error)
s3_minmax22h	1	0	0.5
s3_minmax22v	0.1439	0.1353	0.1396
s3_minmax24	0.1551	0.1422	0.1487
s3_minmax34	0.1637	0.16	0.1618
s3_minmax34h	1	0	0.5
s3_minmax34v	0.1639	0.152	0.1579
s3_minmax41	1	0	0.5
s3_minmax48h	0.1772	0.1564	0.1668
s3_minmax48v	0.1694	0.1684	0.1689
s3_minmax54	0.1806	0.1697	0.1751
***s3_spam14hv***	***0.1328***	***0.1325***	***0.1327***

**Table 4:** Detection results of the EDGE3 × 3 submodels.

Model	*P*_FA_	*P*_MD_	*P*_*E*_ (total error)
s3 × 3_minmax22h	0.1366	0.1962	0.1664
s3 × 3_minmax22v	0.11	0.1802	0.1451
***s3 *×* 3_minmax24***	***0.1302***	***0.1213***	***0.1258***
s3 × 3_minmax41	0.1591	0.1735	0.1663
**s3 **×** 3_spam14hv**	**0.1168**	**0.1323**	**0.1245**

**Table 5:** Detection results of the EDGE5 × 5 submodels and the square submodel.

Model	*P*_FA_	*P*_MD_	*P*_*E*_ (total error)
s5 × 5_minmax22h	0.1656	0.1622	0.1639
**s5 × 5_minmax22v**	**0.1146**	**0.1367**	**0.1256**
s5 × 5_minmax24	0.1639	0.1291	0.1465
s5 × 5_minmax41	0.1882	0.2073	0.1977
***s5 *×* 5_spam14hv***	***0.1131***	***0.114***	***0.1135***
s35_spam11	0.1719	0.1424	0.1572

**Table 6:** Detection results of the merger submodels.

Model	*P*_FA_	*P*_MD_	*P*_*E*_ (total error)
2-best-submodels	0.0959	0.1016	0.0987
4-best-submodels	0.1121	0.1078	0.1100
8-best-submodels	0.1173	0.1162	0.1168

**Table 7:** Detection results on the CASIA V2 dataset.

Model	*P*_FA_ %	*P*_MD_ %	*P*_*E*_ %
YSRM	16.97	9.11	13.04
CrSRM	0.43	0.19	0.31
CbSRM	0.38	0.19	0.28
CrDCT	0.57	0.15	0.36
Cb + DCT	0.16	0.18	0.17
Cr + DCT	0.23	0.19	0.21
[15]	6.70	2.10	4.40
